# Relationships of self-identified cold tolerance and cold-induced vasodilatation in the finger

**DOI:** 10.1186/2046-7648-4-S1-A57

**Published:** 2015-09-14

**Authors:** Joonhee Park, Joo-Young Lee

**Affiliations:** 1 COM:FORT Laboratory, College of Human Ecology, Seoul National University, Seoul, Republic of Korea

## Introduction

Thermal environments in daily life, such as occupational cold exposure and the use of heating facilities and warm clothing, affect acclimatization to both cold and heat. Also, cold tolerance can be cognized by self-identified evaluation. Thermal life-style during daily life might be one of the factors which affect cold-induced vasodilatation (CIVD) when different degrees of thermal stimuli are considered. Therefore, this study investigated whether or not CIVD response is related to self-identified cold and heat tolerances which is attributable to thermal life-style.

## Methods

A self-reported survey and a CIVD test were conducted with 9 males and 34 females. The self-reported questionnaire consisted of 28 questions about personal information and self-identified cold tolerance. Each question used a 4-point scale (1: strongly disagree, 2: disagree, 3: agree, and 4: strongly agree). The CIVD test consisted of 10-min resting, 30-min immersion (3.8(0.3) °C) of the middle finger and 20-min recovery in climatic chamber (27.9(0.1) °C). We used a CIVD definition of >1.0 °C increase in finger skin temperature. As for CIVD variables, the following characteristics from finger skin temperature curves were examined: 1) Time in minutes until the onset of the first CIVD following immersion (t_onset_), 2) finger skin temperature at which the first vasodilation occurred (Minimum finger temperature, T_min_), 3) period in minutes of the temperature rise for the first CIVD (t_peak_), 4) maximum finger skin temperature during the first vasodilation cycle (T_max_), 5) mean finger skin temperature from the moment of the CIVD onset to the end of the cold immersion (T_mean_), 6) difference between T_min _and T_max _(amplitude). Cold resistance index (RI) was calculated using T_mean_, T_min_, and t_onset_.

## Results

By a cluster analysis on the survey results, the participants were divided into two groups: high self-identified cold tolerance (HSCT, n = 25) and low self-identified cold tolerance (LSCT, n = 18). LSCT had lower self-identified cold tolerance, and wore heavier clothing during daily life than HSCT (*P*<0.05). LSCT had significantly lower maximal finger temperature (T_max_), smaller amplitude, and delayed onset time of CIVD when compared to HSCT (*P*<0.05). Some questions for examining the self-identified cold tolerance showed relationships with CIVD variables such as cold tolerance index, T_max_, and amplitude (*P*<0.05).

## Discussion

HSCT had more pronounced and quicker CIVD reactions and higher finger skin temperature when compared to LSCT. This is because HSCT has higher peripheral temperatures during local cold exposure when compared to LSCT. The result of faster t_onset _in HSCT shows that HSCT is the group that has a high level of cold tolerance. This also means that the group classification by self-identified cold tolerance was quite applicable.

## Conclusion

We proved that self-identification during daily life is significantly related to physiological responses. This result suggests that the level of individual cold tolerance can be evaluated by a standardized questionnaire on self-identified thermal tolerance. Such convenient evaluations can be applied to schools or military camps to monitor people who are sensitive to cold on a screening stage of health check-ups.

